# Early-life blood pressure and midlife brain and cognitive health: tests in two birth cohorts

**DOI:** 10.1093/braincomms/fcag172

**Published:** 2026-05-13

**Authors:** Mayibongwe Mugoba, Renate M Houts, Annchen R Knodt, Reremoana F Theodore, Richie Poulton, Ahmad R Hariri, Avshalom Caspi, Terrie E Moffitt, Scott T Chiesa

**Affiliations:** Department of Population Science and Experimental Medicine, Institute of Cardiovascular Science, University College London, London WC1E 7HB, UK; Departments of Psychology and Neuroscience, and Psychiatry and Behavioral Sciences, Duke University, Durham, NC 27708, USA; Departments of Psychology and Neuroscience, and Psychiatry and Behavioral Sciences, Duke University, Durham, NC 27708, USA; Dunedin Multidisciplinary Health and Development Research Unit, University of Otago, Dunedin 9016, New Zealand; Dunedin Multidisciplinary Health and Development Research Unit, University of Otago, Dunedin 9016, New Zealand; Departments of Psychology and Neuroscience, and Psychiatry and Behavioral Sciences, Duke University, Durham, NC 27708, USA; Departments of Psychology and Neuroscience, and Psychiatry and Behavioral Sciences, Duke University, Durham, NC 27708, USA; MRC Social, Genetic, and Developmental Psychiatry Centre, Institute of Psychology, Psychiatry, and Neuroscience, King’s College London, London SE5 8AF, UK; Departments of Psychology and Neuroscience, and Psychiatry and Behavioral Sciences, Duke University, Durham, NC 27708, USA; MRC Social, Genetic, and Developmental Psychiatry Centre, Institute of Psychology, Psychiatry, and Neuroscience, King’s College London, London SE5 8AF, UK; Department of Population Science and Experimental Medicine, Institute of Cardiovascular Science, University College London, London WC1E 7HB, UK

**Keywords:** blood pressure, brain health, cognition, Dunedin, BCS70

## Abstract

Elevated blood pressure (BP) in midlife is a well-established risk factor for impaired brain health and cognitive ability in old age. We hypothesized that exposure to elevated BP within the first five decades of life may contribute to this risk through impacts on brain health and/or cognitive ability evident by midlife. Participants (*n* = 893) were selected from the Dunedin Multidisciplinary Health and Development Study (The Dunedin Study). Exposures were systolic (SBP) and diastolic (DBP) blood pressures measured at ages 7, 11, 18, 26, 32, 38 and 45. Cumulative early-life exposure to blood pressure was also quantified as the area under the curve (AUC). Brain health was assessed at age 45 via imaging measures comprising BrainAGE (difference between chronological age and age predicted from machine-learning models of brain-imaging data), white matter hyperintensity (WMH) burden, and retinal arteriolar calibres (RAC)—a proxy for cerebral small vessel remodelling. Cognitive ability (assessed using IQ) was also measured at age 45, with replication of cognitive findings tested in a larger contemporary cohort, the 1970 British Cohort Study. We found limited evidence for any association between BP in the first four decades of life and brain health or cognitive ability at age 45. Most associations instead emerged for BP from early-midlife onwards. Midlife BP was associated with older BrainAGE (Beta for DBP at age 45 = 0.11 [0.04, 0.19]; *P* = 0.003) and higher WMH burden (Beta = 0.09 [0.02, 0.17]; *P* = 0.019). Effect estimates for SBP were similar. For cognitive ability, DBP at ages 38 and 45 showed modest associations with age 45 IQ, which became null after accounting for childhood IQ. These findings were broadly replicated in the 1970 British Cohort Study for age 47 IQ. Only RAC in The Dunedin Study were found to associate with BP from childhood (Beta for age 7 DBP = −0.09 [−0.16, −0.03]; *P* = 0.006), and the magnitude of these estimates increased during midlife (Beta at age 45 = −0.37 [−0.45, −0.30]; *P* < 0.001). We found little evidence for any association between BP prior to age 40 and BrainAGE, WMH volume, or cognitive ability in midlife. However, cumulative exposure to elevated BP from childhood was associated with reduced RAC, suggesting a potential link between BP and adverse cerebral small vessel remodelling from childhood.

## Teaser

Mugoba *et al.* find little evidence for any association between blood pressure measured across early life and cognitive function, BrainAGE (difference between chronological age and age predicted from machine-learning models of brain-imaging data), or white matter intensity volumes measured in midlife. Associations were found with retinal arteriolar calibres, however, suggesting a potential link between blood pressure and adverse cerebral small vessel remodelling from the earliest years.

## Graphical Abstract

**Graphical Abstract:**
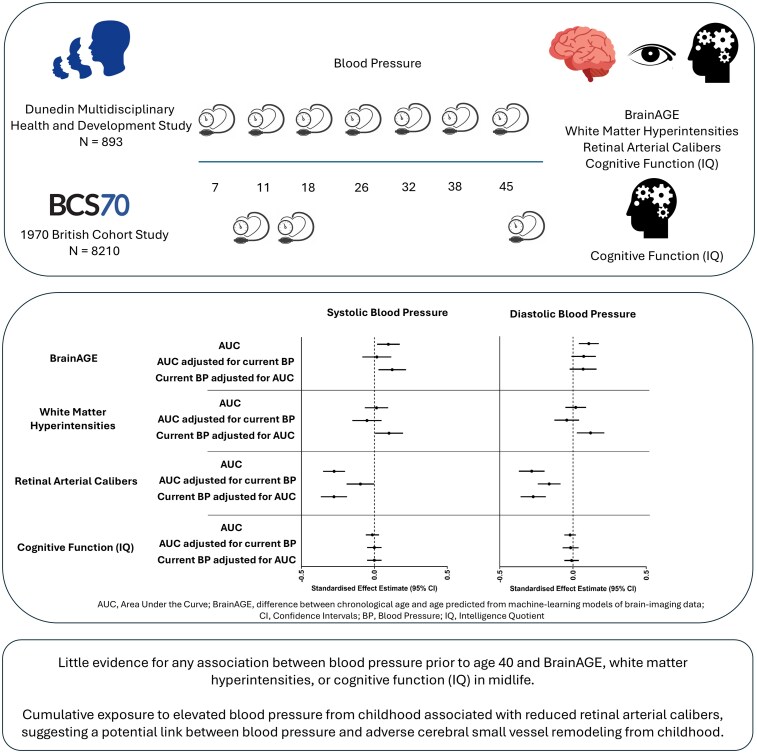

## Introduction

Globally, over 55 million people have dementia, with estimates projecting an increase to at least over 150 million people in 2050.^1^ The World Health Organisation (WHO) has ranked dementia as one of the leading causes of death worldwide, imposing a significant economic and social burden globally with cost estimates surpassing US$1.3 trillion in 2019.^2^

While recent pharmacological trials have seen some success in slowing late-stage cognitive decline,^3,4^ there is a growing appreciation of the potential impact that a population-level approach to lifestyle risk factors may have on future dementia risk. Indeed, recent estimates from the Lancet Commission have suggested that up to 45% of dementias may be caused by 14 common and potentially modifiable risk factors.^5^

One of the most studied of these is elevated blood pressure during midlife, a risk factor which has consistently been shown in observational studies to be a strong predictor of both late-life cognitive impairment and dementia.^6^ These findings have led to suggestions that midlife may therefore represent a particularly sensitive period for dementia risk, where exposure to high blood pressure results in pathophysiological changes within the brain which may impact future cognitive function and decline.^7^

It is well-established, however, that blood pressure tracks across the lifespan, meaning that many individuals with high blood pressure in midlife are likely to have been exposed to its potentially detrimental effects since childhood.^8,9^ Few studies have addressed whether this early or sustained exposure to high blood pressure in the first five decades of life may impact an individual's risk of dementia prior to old age. This early-life risk may manifest through decrements in cognitive ability already established by the midlife period, or through future accelerations in cognitive decline arising from damage to underlying brain health (the latter of which can be assessed through unique imaging-derived indicators such as BrainAGE,^10^ white matter hyperintensity volumes (WMHs),^11^ or retinal measures of microvascular remodelling^12^).

Utilising access to a longitudinal birth cohort followed for almost 50 years, this study aimed to test the hypothesis that cumulative exposure to elevated blood pressure in the early years of life may be associated with lower levels of brain health and/or cognitive ability by midlife. Replication of cognitive findings in a similarly aged but considerably larger cohort of individuals was also sought for comparison to primary analyses.

## Materials and methods

### Study participants

Participants were members of the Dunedin Multidisciplinary Health and Development Study (The Dunedin Study), a longitudinal investigation of health and behaviour in a population-representative birth cohort. The full cohort comprises 1037 individuals (91% of eligible births; 52% male) born between April 1972 and March 1973 in Dunedin, New Zealand, who were eligible based on residence in the province and who participated in the first assessment at age 3 years. The cohort represents the full range of socioeconomic status in the general population of New Zealand's South Island^13^ and matches the New Zealand National Health and Nutrition Survey on adult health indicators (e.g. body mass index (BMI), smoking, physical activity, primary-care visits) and the NZ Census on educational attainment. The cohort is primarily New Zealand European, with 8.6% self-identifying as having Māori ethnicity at age 45. Assessments in this study were carried out at birth and ages 3, 5, 7, 9, 11, 13, 15, 18, 21, 26, 32, 38 and 45 years, when 94.1% (*N* = 938) of the 997 participants still alive took part. 875 (93% of age-45 participants) also completed MRI scanning. Scanned participants did not differ from other living participants on childhood Socioeconomic Status (SES) or childhood Intelligence Quotient (IQ) (see attrition analysis in Supplementary Fig. 1). The Dunedin Study was approved by the New Zealand Health and Disability Ethics Committee. Participants gave written informed consent.

### Early-life blood pressure exposures

Blood pressure measures were available from childhood through to adulthood at ages 7, 11, 18, 26, 32, 38, and 45. Blood pressure up to 18 years was measured using a London School of Hygiene and Tropical Medicine blind mercury sphygmomanometer (Cinetronics Ltd, Mildenhall, United Kingdom). Thereafter, a Hawksley random-zero sphygmomanometer (Hawksley and Sons Ltd, Sussex, United Kingdom) was used for 45 years, followed by an automatic BpTRU Vital Signs Monitor BPM 200 (BpTRU Medical Devices, Canada). Blood pressure was measured at all time-points in the seated position and reported as the average of 2–3 measurements taken approximately 5 mins apart.

### Mid-life imaging outcomes at age 45

Three phenotypes assessing grey matter structure (BrainAGE; difference between chronological age and age predicted from machine-learning models of brain-imaging data), white matter damage (WMH), and cerebrovascular small vessel remodelling (proxied through retinal arteriolar calibres) were chosen as outcomes. All three measures have previously been demonstrated to be associated with risk factors in young adulthood to midlife^12,14,15^ and also to be associated with dementia-related complications in later life.^16-18^ Of the 875 scanned participants who had at least one MRI scan, 867 had both a T1 and a FLAIR image, which are required to extract WMHs. Eight participants were removed for excessive false positives, four because they had incidental findings that interfered with the calculating algorithm, three for having multiple sclerosis, and nine for missing IQ data in childhood or adulthood, leaving 843 participants for the final analysis.

#### Image acquisition

A Siemens Skyra 3T scanner equipped with a 64-channel head/neck coil was used to scan each participant at the Pacific Radiology Imaging Centre in Dunedin, New Zealand. High-resolution structural images were obtained using a T1-weighted MP-RAGE sequence with the following parameters: Repetition Time (TR) = 2400 ms; Echo Time (TE) = 1.98 ms; 208 sagittal slices; flip angle = 9°; Field of View (FOV) = 224 mm; matrix = 256 × 256; slice thickness = 0.9 mm with no gap (voxel size 0.9 mm × 0.875 mm × 0.875 mm). Total scan time for these measures was 6 min and 52 s. 3D FLAIR images were also obtained using the following parameters: TR = 8000 ms; TE = 399 ms; 160 sagittal slices; FOV = 240 mm; matrix = 232 × 256; slice thickness = 1.2 mm (voxel size 0.9 mm × 0.9 mm × 1.2 mm). Total scan time for these measures was 5 min and 38 s.

#### BrainAGE

The methodology behind the calculation of BrainAGE has been previously described in detail.^10^ Briefly, a previously developed and publicly available algorithm was used to predict chronological age based on multiple measures of brain structure derived from Freesurfer v5.3. Specifically, the algorithm was trained on vertex-wise cortical thickness and surface area extracted from fsaverage4 standard space and subcortical volumes extracted from aseg parcellation. Test-retest reliability was assessed in 20 study members (mean interval between scans = 79 days) and was shown to be excellent (intraclass correlation coefficient (ICC) [95%CI] = 0.81 [0.59, 0.92]; *P* < 0.001). This technique has also previously been shown to predict chronological age in independent samples as well as to relate to age-related cognitive impairment in later life. All regression analyses use BrainAGE scores (i.e. brain age gap estimate, or the difference between an individual’s predicted age from MRI data and their exact chronological age, between birth and the date of the MRI scan).

#### White matter hyperintensities (WMH)

WMH volumes for each participant were quantified using T1-weighted and FLAIR images. WMHs were identified using UBO Detector, a cluster-based, fully-automated pipeline employing FMRIB’s Automated Segmentation Tool^11^ to identify candidate clusters. A K-nearest neighbour algorithm was employed to classify images as WMHs or non-WMHs based on intensity, anatomical location, and cluster size features. A Diffeomorphic Anatomical Registration through Exponentiated Lie (DARTEL) template of 55 years or younger was used to best approximate the age of the cohort,^11^ and a grey matter mask was applied to decrease the chance of false positives. The resulting WMH probability maps were thresholded at the suggested standard of 0.7.^11^ The UBO pipeline was chosen because of its high reliability in our data (test-retest ICC = 0.87) and its out-of-sample performance.^11^ For additional quality assurance, however, every participant’s UBO-generated WMH map was visually inspected to check for false positives (e.g. areas such as the septum that appear similar to WMHs on FLAIR images).

#### Retinal arteriolar calibre

Digital fundus photographs were taken after 5 min of dark adaptation at the Dunedin Research Unit using a Canon NMR-45 with a 20D single-lens reflex backing (Canon, Tokyo, Japan). Photographs of both eyes were graded at the Singapore Eye Research Institute using semi-automated software (Singapore I Vessel Assessment [SIVA] v3.0), and an average for both eyes was calculated. Trained graders, blind to participants' characteristics, used the SIVA program to measure the retinal arteriolar calibres (i.e. lumen diameters) according to a standardized protocol with high intergrader reliability.^19^ Measurements were made for arterioles where they passed through a region located 0.5 to 2 disk diameters from the optic disk margin. Calibres were based on the six largest arterioles passing through this region and were summarized as a central retinal artery equivalent (CRAE) using the revised Knudtson-Parr-Hubbard formula.^20,21^ All scores were subsequently corrected for venous calibre and sex prior to inclusion in models.

### Mid-life cognitive outcomes

Cognition at age 45 was assessed with the Wechsler Adult Intelligence Scale (WAIS-IV), which measures IQ by summarising four domains of cognitive function: Verbal Comprehension, Perceptual Reasoning, Working Memory, and Processing Speed. Tests were conducted by trained psychometrists using standard protocols.

### Covariates

Covariates were sex, age-45 BMI, childhood IQ, and the highest education level achieved. Childhood IQ was measured at ages 7, 9 and 11 using the Wechsler Intelligence Scale for Children–Revised (WISC-R), prorated from eight subtests: Information, Similarities, Vocabulary, Block Design, Object Assembly, Picture Completion, Arithmetic and Digit Symbol tasks. The three total IQ measures were subsequently averaged across all ages to provide a single metric for childhood IQ.

### Replication of cognitive analyses in a secondary cohort

Replication of cognitive findings from The Dunedin Study was also sought in a similarly aged but considerably larger sample of individuals recruited to the 1970 British Cohort Study (*n* = 8210).^22^ Full details on the methodology employed within this cohort can be found in the Supplementary material. In brief, exposures were BP measured in childhood (age 10), adolescence (age 16), and midlife (age 47), and the outcome was cognitive ability at age 47 (IQ derived from tests of immediate and delayed recall, executive function, and processing speed).

### Statistical analysis

Continuous descriptive data were summarized as mean (SD). Categorical data were summarized as % if binary or by category if >2 groups. Multiple linear regression models were used to test associations between a range of BP exposures and cognitive and neuroimaging measures. First, SBP or DBP at ages 7, 11, 18, 26, 32, 38 and 45 were tested against neuroimaging and cognitive outcomes at age 45 using the following levels of adjustment: Model 1 = Adjustments for sex and current BMI; Model 2 = Model 1 + further adjustments for educational attainment and childhood IQ. Next, the area under the curve (AUC) between contiguous timepoints was calculated for each BP trait using trapezoid sums and summed to represent cumulative BP exposure during childhood and adolescence (ages 7–26), young adulthood to midlife (ages 26–45), and across the whole of early life (ages 7–45). The same regression models as described before were then re-run with AUC as the exposure, before additionally running with a 3rd model, further adjusting for age-45 BP to separate the independent effects of cumulative versus contemporary blood pressure exposures. All results are reported as standardized beta estimates (i.e. SD change in outcome per SD change in exposure) to allow for comparison between outcomes. In line with previous analyses in The Dunedin Study, multiple imputation was used to impute missing exposure data in study participants who had at least 4 BP measurements across the 7 assessment times. All statistical analyses were performed in SAS V9.4 (TS Level 1M8). An *a-priori* decision was made to interpret findings mainly on the basis of model estimates and their 95% CIs rather than assign ‘significance’ using arbitrary standard (0.05) or FDR-corrected *P* value cutoffs. All *P*-values are still highlighted throughout, however, for reference.

## Results

Participant characteristics for the Dunedin Study are in Table 1. Characteristics for the 1970 British Cohort Study secondary replication cohort are in the Supplementary Table 1. Correlations between outcome measures in The Dunedin Study at age 45 can be seen in Supplementary Table 2.

**Table 1:** Participant characteristics

	*N*	% or M (SD)
*Demographics*		
Sex (% female)	893	49%
Current BMI (kg/m^2^)	893	28.4 (5.8)
*Early-Life Factors*		
Childhood IQ (score)	893	101 (14)
Highest Educational Level Attained (%)	893	
No Certification	122	13.7%
School Certification	128	14.3%
HS Grad Equivalent	363	40.7%
BA or Higher	280	31.4%
*Exposures*		
Systolic Blood Pressure (mmHg)		
Age 7	893	102 (7)
Age 11	893	103 (7)
Age 18	893	121 (11)
Age 26	893	117 (11)
Age 32	893	118 (12)
Age 38	893	120 (12)
Age 45	893	121 (15)
Diastolic Blood Pressure (mmHg)		
Age 7	893	61 (6)
Age 11	893	68 (7)
Age 18	893	57 (9)
Age 26	893	72 (9)
Age 32	893	76 (9)
Age 38	893	78 (10)
Age 45	893	80 (10)
*Outcomes*		
BrainAGE (difference from biological age in years)	848	−0.13 (7.83)
Ln-transformed White Matter Hyperintensities (mm^3^)	834	6.52 (0.79)
Retinal Arteriolar Calibre (standardized score)	868	−0.01 (1.00)
IQ (score)	891	99 (15)

Data are mean ± SD, or %. BMI, body mass index; SBP, systolic blood pressure; DBP, diastolic blood pressure; WMH, white matter hyperintensities; IQ, intelligence quotient.

### Imaging outcomes

#### BrainAGE

**Figure 1:**
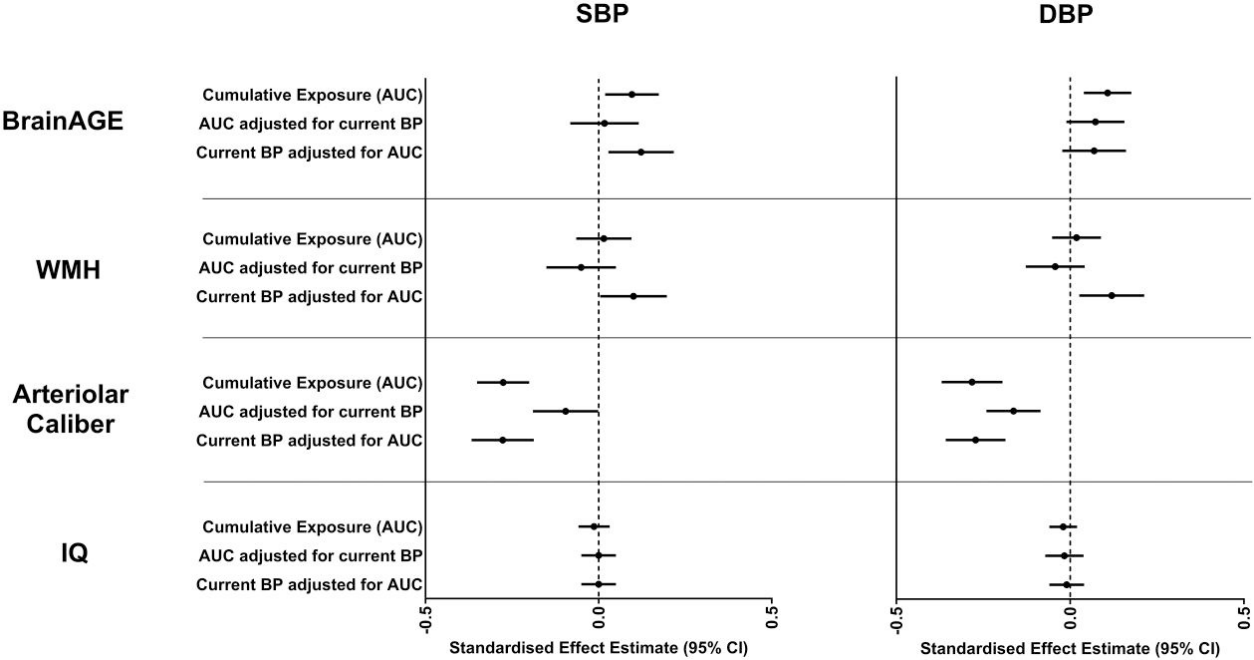
**Association between cumulative versus current blood pressure exposures and midlife brain health and cognitive ability.** Data are displayed as standardized effect estimates (Beta) and 95% CIs obtained from multivariable linear regression models with the following adjustments: sex, current BMI, childhood IQ, and highest educational attainment. Area under the curve (AUC) between contiguous timepoints was calculated for each BP trait using trapezoidal sums and summed to represent cumulative BP exposure across ages 7–45. Due to the known tracking of blood pressure across the life course, associations between AUC exposures and all outcomes were additionally run both with and without adjustments for current blood pressure to separate the independent effects of cumulative versus contemporary blood pressure exposures. All outcomes were measured at age 45, comprising BrainAGE (*n* = 848), white matter hyperintensities (*n* = 834), retinal arteriolar calibre (*n* = 868), and IQ (*n* = 891). BrainAGE, difference between chronological age and age predicted from machine-learning models of brain-imaging data; BMI, body mass index; BP, blood pressure; SBP, systolic blood pressure; DBP, diastolic blood pressure; WMH, white matter hyperintensities; IQ, intelligence quotient.; CI, confidence intervals.

We found little evidence of any association between either SBP or DBP in the first three decades of life and BrainAGE in midlife. Associations did begin to emerge for BP assessed from the age of 32 years onwards, supporting previous claims of this early midlife period as a potentially important time linking BP and cerebral traits (Table 2). Cumulative BP exposure across the early decades of life was associated with BrainAGE even after adjustments for other early-life factors (i.e. childhood IQ and educational attainment), but adjustment for age-45 BP measures attenuated these associations towards the null, suggesting a predominantly contemporary association of BP with age-45 BrainAGE (Fig. 1 and Supplementary Tables 3 and 4).

**Table 2:** Association between early-life blood pressure exposures and midlife brain health and cognitive ability

Standardized Effect Estimate (β), (95% Confidence Intervals), *P*-value								
	BrainAGE		WMH Volume		Arteriolar Caliber		IQ	
	SBP	DBP	SBP	DBP	SBP	DBP	SBP	DBP
Age 7								
Model 1	0.03 (−0.04, 0.10) 0.411	0.03 (−0.04, 0.10) 0.364	0.04 (−0.03, 0.11) 0.292	−0.04 (−0.11, 0.02) 0.205	**−0.10 (−0.16, −0.03)** **0.004**	**−0.09 (−0.16, −0.03)** **0.006**	−0.02 (−0.08, 0.05) 0.590	−0.02 (−0.08, 0.05) 0.599
Model 2	0.03 (−0.04, 0.10) 0.400	0.03 (−0.03, 0.10) 0.306	0.04 (−0.03, 0.11) 0.268	−0.04 (−0.11, 0.03) 0.219	**−0.10 (−0.16, −0.03)** **0.004**	**−0.09 (−0.16, −0.03)** **0.006**	−0.03 (−0.07, 0.01) 0.218	−0.04 (−0.08, 0.00) 0.073
Age 11								
Model 1	0.06 (−0.01, 0.13) 0.075	0.03 (−0.03, 0.10) 0.339	0.01 (−0.05, 0.08) 0.695	0.04 (−0.03, 0.10) 0.302	**−0.15 (−0.22, −0.09)<0.001**	**−0.15 (−0.21, −0.08)<0.001**	0.01 (−0.05, 0.08) 0.736	−0.06 (−0.13, 0.00) 0.064
Model 2	**0.07 (0.00, 0.14)** **0.041**	0.03 (−0.04, 0.09) 0.455	0.02 (−0.05, 0.09) 0.548	0.03 (−0.04, 0.10) 0.382	**−0.15 (−0.22, −0.09)<0.001**	**−0.15 (−0.21, −0.08)<0.001**	−0.02 (−0.06, 0.02) 0.294	−0.03 (−0.07, 0.01) 0.163
Age 18								
Model 1	0.05 (−0.03, 0.13) 0.212	0.04 (−0.02, 0.11) 0.218	−0.06 (−0.14, 0.02) 0.154	−0.06 (−0.12, 0.01) 0.110	**−0.08 (−0.16, 0.00)** **0.048**	**−0.10 (−0.16, −0.03)** **0.003**	−0.04 (−0.11, 0.04) 0.365	0.01 (−0.05, 0.08) 0.683
Model 2	0.05 (−0.03, 0.13) 0.236	0.05 (−0.02, 0.11) 0.168	−0.05 (−0.13, 0.03) 0.186	−0.05 (−0.12, 0.02) 0.143	**−0.08 (−0.16, 0.00)** **0.049**	**−0.10 (−0.17, −0.03)** **0.003**	−0.02 (−0.07, 0.03) 0.376	0.00 (−0.04, 0.04) 1.00
Age 26								
Model 1	0.02 (−0.05, 0.10) 0.538	0.04 (−0.03, 0.10) 0.289	−0.03 (−0.10, 0.05) 0.508	0.01 (−0.06, 0.07) 0.867	**−0.19 (−0.26, −0.12)<0.001**	**−0.19 (−0.26, −0.13)<0.001**	0.04 (−0.03, 0.11) 0.297	0.03 (−0.03, 0.10) 0.339
Model 2	0.03 (−0.04, 0.11) 0.415	0.05 (−0.02, 0.12) 0.145	−0.02 (−0.10, 0.06) 0.591	0.01 (−0.05, 0.08) 0.674	**−0.19 (−0.26, −0.12)<0.001**	**−0.20 (−0.26, −0.13)<0.001**	0.00 (−0.04, 0.05) 0.860	−0.02 (−0.06, 0.02) 0.250
Age 32								
Model 1	0.03 (−0.04, 0.11) 0.361	**0.08 (0.01, 0.15)** **0.018**	0.01 (−0.07, 0.08) 0.855	0.00 (−0.07, 0.07) 0.945	**−0.18 (−0.25, −0.11)<0.001**	**−0.17 (−0.24, −0.11)** **< 0.001**	0.02 (−0.05, 0.10) 0.514	0.03 (−0.03, 0.10) 0.322
Model 2	0.04 (−0.04, 0.11) 0.297	**0.09 (0.02, 0.16)** **0.008**	0.01 (−0.06, 0.09) 0.757	0.00 (−0.07, 0.07) 0.904	**−0.18 (−0.25, −0.11)** **< 0.001**	**−0.17 (−0.24, −0.11)<0.001**	0.01 (−0.04, 0.05) 0.779	0.01 (−0.03, 0.05) 0.727
Age 38								
Model 1	**0.10 (0.03, 0.17)** **0.008**	**0.13 (0.05, 0.20)** **0.001**	0.04 (−0.04, 0.11) 0.321	0.05 (−0.02, 0.13) 0.146	**−0.24 (−0.31, −0.17)<0.001**	**−0.30 (−0.37, −0.23)<0.001**	−0.01 (−0.08, 0.06) 0.764	**−0.09 (−0.16, −0.02)** **0.015**
Model 2	**0.10 (0.03, 0.18)** **0.005**	**0.12 (0.05, 0.19)** **0.001**	0.04 (−0.03, 0.12) 0.257	0.05 (−0.02, 0.13) 0.147	**−0.24 (−0.31, −0.17)<0.001**	**−0.30 (−0.37, −0.24)<0.001**	−0.01 (−0.06, 0.03) 0.518	−0.02 (−0.06, 0.02) 0.344
Age 45								
Model 1	**0.14 (0.07, 0.22)<0.001**	**0.12 (0.05, 0.20)** **0.002**	0.08 (0.00, 0.15) 0.050	**0.10 (0.02, 0.18)** **0.011**	**−0.33 (−0.40, −0.26)<0.001**	**−0.37 (−0.44, −0.30)<0.001**	−0.04 (−0.12, 0.03) 0.240	−0.07 (−0.14, 0.01) 0.090
Model 2	**0.13 (0.06, 0.21)** **0.001**	**0.11 (0.04, 0.19)** **0.003**	0.07 (−0.01, 0.15) 0.068	**0.09 (0.02, 0.17)** **0.019**	**−0.33 (−0.40, −0.26)<0.001**	**−0.37 (−0.45, −0.30)<0.001**	−0.01 (−0.06, 0.03) 0.609	−0.03 (−0.07, 0.02) 0.277

Data are displayed as standardized effect estimates (Beta), 95% Confidence Intervals (CI) and *P*-values obtained from multivariable linear regression models with the following adjustments: Model 1 = Adjustments for sex and current BMI; Model 2 = Model 1 + further adjustments for childhood IQ and highest educational attainment. All outcomes were measured at age 45, comprising BrainAGE (*n* = 848), white matter hyperintensities (*n* = 834), retinal arteriolar calibre (*n* = 868), and IQ (*n* = 891). BMI, body mass index; SBP, systolic blood pressure; DBP, diastolic blood pressure; WMH, white matter hyperintensities; IQ, intelligence quotient. Bold text indicates *P* < 0.05.

#### White matter hyperintensities

We found little to no evidence of any association between BP prior to age 45 and WMH volumes in midlife, either when looking at individual BP timepoints measured across the early decades of life (Table 2) or when assessing BP exposure as a cumulative burden (Fig. 1 and Supplementary Tables 3 and 4). However, both SBP and DBP at age 45 were associated with contemporary WMHs (∼8% increase in WMH volume per 10 mmHg increase in SBP), even after accounting for other early-life factors and cumulative BP exposure over time.

#### Retinal arteriolar calibre

SBP and DBP at every time point in early life were associated with decreased arteriolar calibres in midlife, and the magnitude of these negative associations progressively increased (e.g. standardized beta of roughly −0.10 at age 7 to −0.33 at age 45 (Table 2)). Both AUC and contemporary BP measures were independently associated with arteriolar diameters when included in the same model, suggesting contributions from both cumulative exposure and current BP levels to arteriolar tone and remodelling (Fig. 1 and Supplementary Tables 3 and 4).

### Cognitive outcomes

We found minimal evidence for any association between blood pressure measured in the early decades of life and cognitive function assessed in mid-life (Table 2). We did, however, find weak associations between DBP assessed at age 38 onwards and cognitive ability (e.g. Beta for DBP at age 38 = −0.09 [−0.16, −0.02]; *P* = 0.015), although these effects attenuated towards the null after adjustment for early-life differences in IQ and education (Beta = −0.02 [−0.06, 0.02]; *P* = 0.344).

### Replication of cognitive analyses in the secondary cohort

Cognitive findings from The Dunedin Study were broadly replicated in the larger 1970 British Cohort Study. Once again, little evidence was found for any associations between blood pressure measured in childhood (age 10) or adolescence (age 16) and cognitive function measured in midlife (Supplementary Table 5). Similar to The Dunedin Study, we found associations between BP and IQ only in the midlife period (Beta for cross-sectional analyses at age 47 years = −0.08 [−0.11, −0.05]; *P* < 0.001). As in The Dunedin Study, these estimates are attenuated after accounting for early-life factors (Beta = −0.04 [−0.07, −0.01]; *P* = 0.018).

## Discussion

Our study found little evidence for any associations between blood pressure in the earliest decades of life and brain outcomes, and cognitive ability tested at age 45. Instead, the majority of associations emerged for BP assessed closer to midlife. Blood pressure from around age 40 onwards was found to be associated with higher BrainAGE and WMH burden, alongside weaker evidence for lower IQ. The exception to this pattern was for retinal arteriolar calibers (a proxy for cerebral small vessel remodelling), which demonstrated negative associations with blood pressure assessed from as early as age 7. These associations strengthened over time, suggesting a possible increasingly detrimental effect of cumulative early-life blood pressure exposure on the small vessels of the brain.

Dementia is almost exclusively a disease of older adults, and most studies linking blood pressure to dementia risk therefore focus on the mid-to-late life period. Numerous observational studies at this age have repeatedly demonstrated associations between elevated blood pressure in midlife and brain health,^23^ cognitive function,^6^ and risk of incident dementia^24^ in later life. Furthermore, findings from the recent SPRINT-MIND trial have provided the first evidence linking intensive blood pressure lowering at age 50+ to a reduced risk of future mild cognitive impairment and probable dementia.^25^ As such, midlife blood pressure is now widely considered to be one of the major modifiable risk factors for dementia, and is estimated in the Lancet 2024 Dementia Commission Report to potentially account for ∼2% of the total population attributable risk for the disease.^5^

Associations between blood pressure and brain and cognitive ability early in the lifespan remain poorly studied, despite the known tracking of blood pressure from childhood and known links between blood pressure and impaired cognitive function in the midlife period. We hypothesized that exposure to higher blood pressure in the early years of life would be associated with detrimental subclinical differences in brain and cognitive ability that are already evident by midlife, similar to that shown in other vital organs within the human body over this timeframe.^26,27^ The data were largely inconsistent with our hypothesis.

For cognitive outcomes, we saw little evidence for any association between elevated blood pressure across the early decades of life and cognition in midlife; findings which contrast with some^28-33^—but not all^34,35^—studies. With regard to the former, numerous findings from both the Cardiovascular Risk in Young Finns study and Coronary Artery Risk Development in Young Adults (CARDIA) study have suggested seemingly detrimental associations between blood pressure in the early decades of life and various measures of cognitive function measured in midlife.^28-33^ A number of methodological differences between these studies and our own may speculatively explain observed differences. Firstly, while all studies from both CARDIA and Young Finns cohorts attempted to control for potential confounding bias via careful adjustment for a range of social and biological covariates of interest, all were limited by an absence of any measure of preceding childhood cognitive ability—a highly stable trait that is itself predictive of poor cardiovascular health across the lifespan.^36^ The potential, therefore, exists for any observed relationships in these cohorts to possibly represent confounding via reverse causality, whereby a consistently low IQ across the first 5–6 decades of life results in a higher risk of elevated blood pressure by midlife, rather than the other way round. Indeed, this phenomenon has recently been demonstrated when interpreting early-life associations linking excess bodyweight to midlife cognitive ability,^37^ with life-course trajectories of obesity in children with low cognitive function found to closely resemble those often interpreted to be operating in the opposite direction.^29^ It should be noted that adjustment for childhood cognitive ability had little effect on current early-life estimates reported here, suggesting that early-life cognitive function was unlikely to be a major source of bias when assessing associations with childhood/adolescent blood pressure. A greater attenuating effect was found when looking at associations with later measures of blood pressure at age 45, raising the potential for reverse causality to at least in part explain observed later-life associations. Secondly, both the CARDIA and Young Finns studies assessed cognitive outcomes across multiple cognitive domains rather than a marker of overall cognition (IQ) measured here. In addition to the methodological differences, a closer examination of the outcomes in the CARDIA and Young Finns studies reveals some nuance in the results. In the Young Finns study, the association between blood pressure and cognition was only observed with systolic blood pressure, while diastolic blood pressure showed no association across cognitive domains. Similarly, in the CARDIA study, while systolic blood pressure demonstrated an association with cognition across the measured cognitive domains, the association with diastolic blood pressure was found to be inconsistent.

With respect to neuroimaging outcomes, our data provide some evidence for an association between cumulative early-life blood pressure exposures and midlife BrainAGE and retinal arteriolar diameters. Both of these measures have previously been associated with an increased risk of dementia in long-term follow-up,^16,17^ suggesting that—despite a lack of convincing association with cognitive function at this early age—elevated early-life blood pressure may still putatively increase risk for later-life dementia through changes in underlying brain structure and physiology. For diastolic blood pressure, these associations remained broadly unchanged even after adjustment for midlife blood pressure levels, suggesting that long-term exposure to steady-state elevations in blood pressure across the early decades may lead to adverse remodelling within the small vessels of the brain that can emerge from as early as childhood. This microcirculatory remodelling response to hypertension is well-established in the literature, with chronic vasoconstriction over time known to result in an inward remodelling of vessels in an attempt to protect the fragile microcirculation from increased systemic pressures.^38^ Reductions in retinal arteriolar diameters have previously been shown to manifest in children as young as 6 years old,^12^ and are now shown in the current study to persist and strengthen over time until midlife. Interestingly, this association was not observed for WMH burden, with concurrent blood pressure levels at age 45 the only exposure found to associate with this presumed marker of overt vascular damage. The timing of this emerging association coincides with a period in the life-course in which blood pressure phenotypes are known to change, with a widening blood pressure distribution at a population level, an increasing prevalence of hypertension, and an emerging stiffening of the major arteries resulting in an increased impact of systolic and pulsatile pressures on end-organ damage.^39^ These changes in blood pressure phenotypes have been shown in multiple studies to relate to future cerebral damage and cognitive impairment, with the underlying putative mechanisms believed to be linked to small vessel damage resulting from repeated pulsatile stress.^40^ Our finding of small vessel remodelling in individuals exposed to decades of elevated diastolic blood pressure during early-life, and the subsequent possible emergence of white matter hyperintensities in association more severe systolic blood pressure exposures in midlife, raises the possibility that a reduction in cerebral small vessel ‘reserve’ during early life may predispose individuals to augmented negative effects of increasing systolic and pulse pressures as they enter the midlife period.

This study has a number of strengths and limitations. Firstly, the inclusion of two large contemporary, population-based birth cohorts of near identical ages allowed us greater power to investigate both cognition and neuroimaging while minimising the risk of secular cohort effects. The availability of MRI data in only the Dunedin Study and at only one timepoint, however, did not allow the tracking of longitudinal changes in neuroimaging outcomes. Secondly, a lack of blood pressure measures through the 3rd to 5th decades of life in the 1970 British Cohort Study prevented associations between cumulative exposure to blood pressure and cognitive function from being tested in the same way as done for neuroimaging outcomes. Thirdly, the countries from which each cohort was recruited, and the time of recruitment (1970s), means that both cohorts are comprised of predominantly white individuals, and findings may not be generalisable to other ethnicities. Fourthly, while our findings did not provide any strong evidence for an association between early-life blood pressure exposure and midlife dementia-related biomarkers, it is currently not possible to test whether early-life blood pressure shows any association with later-life risk. Continued follow-up of both of these life-course cohorts will allow this to be tested in time. Finally, as with all observational studies, the risk of unmeasured confounding and other biases means that any associations reported here should not be interpreted as causal.

In conclusion, we found little evidence for any association between midlife cognitive function, BrainAGE, or white matter intensity volume and blood pressure prior to the fourth decade of life. Cumulative exposure to elevated blood pressure from as early as age 7 was found to be associated with reduced retinal arteriolar calibres, however, suggesting a potential link between blood pressure exposure and adverse cerebral small vessel remodelling from the earliest years.

## Supplementary Material

fcag172_Supplementary_Data

## Data Availability

Details on accessing data from The Dunedin Study can be found in the Policy Statement and Code of Practice document available at https://dunedinstudy.otago.ac.nz/. 1970 British Cohort Study data are available from the UK Data Service repository at https://ukdataservice.ac.uk. All statistical code related to this paper is available in an open-access GitHub repository located at https://github.com/scottchiesa/early-life-bp-cog-brain.
