# Supplementary material for: Early-life blood pressure and midlife brain and cognitive health: tests in two birth cohorts
Source: Brain Commun. 2026 May 13;8(3):fcag172. doi: 10.1093/braincomms/fcag172 (PMC13218384; doi:10.1093/braincomms/fcag172)
Supplement: fcag172_Supplementary_Data [file fcag172_supplementary_data.docx]

**SUPPLEMENTARY MATERIAL**

**EARLY-LIFE BLOOD PRESSURE AND MIDLIFE BRAIN AND COGNITIVE HEALTH: TESTS IN TWO BIRTH COHORTS**

**Mugoba et al 2026**

# TABLE OF CONTENTS

[TABLE OF CONTENTS 2](#_Toc229389769)

[SUPPLEMENTARY METHODS 3](#_Toc229389770)

[Replication of Cognitive Analyses in the 1970 British Cohort Study 3](#_Toc229389771)

[Study Participants 3](#_Toc229389772)

[Early-life blood pressure exposure 3](#_Toc229389773)

[Midlife cognitive outcomes BCS70 4](#_Toc229389774)

[Covariates 5](#_Toc229389775)

[Statistical Analysis 6](#_Toc229389776)

[SUPPLEMENTARY TABLES 7](#_Toc229389777)

[Supplementary Table 1: BCS70 Participant Characteristics 7](#_Toc229389778)

[Supplementary Table 2: Correlations between outcome measures at age 45 in the Dunedin Study 8](#_Toc229389779)

[Supplementary Table 3: Associations between Cumulative Systolic Blood Pressure Exposures during Childhood/Adolescence and Young Adulthood/Midlife with Midlife Brain Health and Cognitive Ability in the Dunedin Study 9](#_Toc229389780)

[Supplementary Table 4: Associations between Cumulative Diastolic Blood Pressure Exposures during Childhood/Adolescence and Young Adulthood/Midlife with Midlife Brain Health and Cognitive Ability in the Dunedin Study 11](#_Toc229389781)

[Supplementary Table 5: Associations between blood pressure measured in childhood, adolescence, and midlife and cognitive function measured in midlife in BCS70 13](#_Toc229389782)

[SUPPLEMENTARY FIGURE 14](#_Toc229389783)

[Supplementary Figure 1 - Attrition analyses in the Dunedin Study 14](#_Toc229389784)

[SUPPLEMENTARY REFERENCES 16](#_Toc229389785)

# SUPPLEMENTARY METHODS

## Replication of Cognitive Analyses in the 1970 British Cohort Study

### Study Participants

Participants were members of the 1970 British Cohort Study, an ongoing multidisciplinary longitudinal study, which tracked the lives of over 17,000 individuals born in the UK during a single week in 1970, gathering comprehensive health, social and economic data^1^. The cohort has undergone nine sweeps of data collection from birth through ages 5, 10, 16, 26, 30, 34, 38, 42, 46(N=8581), with the latest concluding in 2024 at age 51-53. Selective attrition resulted in more cohort members from advantaged socioeconomic backgrounds (27.1% non-manual paternal social class at birth, 32.2% at age 46) and more women (48.2% at birth, 52.0% at age 46). The original cohort distribution, however, has been shown to be restored using observed variables as auxiliary variables in analyses. Ethics approval was obtained from the National Health Service Research Ethics Committee in advance of each sweep of data collection.

### Early-life blood pressure exposure

In the BCS70 study, blood pressure (BP) measurements were recorded at ages 10,16 and 46. Blood pressure at ages 10 and 16 years was taken using a mercury sphygmomanometer, and at 46 years with an automated Omron HEM-907 monitor. All readings were taken in a quiet room with the participants seated and the appropriate cuff size was used for each reading.

### Midlife cognitive outcomes BCS70

Cognition was assessed in BCS70 in mid-life (age 45-46) using a battery of four separate cognitive tests comprising immediate recall, delayed recall, verbal fluency and processing speed. Immediate and delayed recall: One of four lists of 10 common words were selected by the interviewer and presented to participant via a recorded voice at a rate of one word every 2-seconds. After the list had been read out, the participants were given two minutes to recall as many of the words as possible (in no particular order). The total number recalled was entered into the computer by the interviewer. After additional tests were administered (animal naming and letter cancellation), the interviewer asked the participant to again recall as many words as possible from the original list (words not repeated by interviewer). This delayed memory task was administered approximately five minutes after the initial recall task. Scores for each test ranged from 0-10 based on the number of words recalled. For verbal fluency, participants were asked to name as many different animals as possible within a one-minute timeframe then given a score based on the total number of animals named. Processing speed was assessed by a timed letter search and cancellation test. Participants were given a page of random letters arranged in rows (N = 26) and columns (N = 30) and were asked to cross out as many “Ps” and “Ws” as possible within a one-minute timeframe. They were instructed to work across each row from left-to-right as if they were reading a page and they were asked to perform the task as quickly and accurately as possible. Once the allotted time was over, the respondent was asked to underline the last letter that reached their eye (any letter, target or otherwise). The total number of letters searched was summed to provide a measure of speed of processing.

Next, the four cognitive tests underwent principal component analysis (PCA) to derive a single measure capturing overall cognition. PCA transforms multidimensional data into lower-dimensional representations while retaining as much information as possible. The first principal component captured the highest level of variance common to all four tests (immediate recall, delayed recall, executive function, and processing speed), providing a standardized measure of general cognition analogous to the underlying general intelligence factor that is captured by the intelligence quotient (IQ).

### Covariates

Covariates included sex, childhood overcrowding, childhood socioeconomic factors, childhood cognitive ability, adult socioeconomic factors, highest adult education, body mass index, smoking status and history, malaise and physical activity. Childhood cognitive ability was based on principal component analysis of four cognitive tests: Friendly Maths Test, Language Comprehension Test, Maths British Ability Skills and Matrices British Ability Skills. Childhood overcrowding was based on average number of persons per room during childhood. Childhood socioeconomic factors were based on the father’s occupational status when the child was aged ~10 years old and classified using the 1990 Registrar General’s Social Class system: professional, managerial and technical, skilled non-manual, skilled manual, partly skilled and unskilled. Adult socioeconomic factors were based on contemporary occupational activity, classified according to the United Kingdom National Statistics Socio-economic Classification (NS-SEC) and modelled as three categories – Managers, Intermediate, and Lower/Technical.

### Statistical Analysis

In BCS70, multiple linear regression was used to test the association between BP exposures in childhood, adolescence and adulthood and cognition in mid-life using three models: Model 1 = adjustment for age, sex, and ethnicity; Model 2 = Model 1 + further adjustment for early life factors (childhood cognitive ability, childhood overcrowding and childhood socioeconomic status); Model 3 = Model 2 + further adjustment for mid-life factors (highest adult education, adult socioeconomic status, adult BMI, smoking status, malaise, and physical activity). Multiple imputation (50 imputations) was performed for all missing values in the variables in the datasets following guidance set out by the Centre for Longitudinal Studies. All statistical analyses were performed in Stata 17 (StataCorp 2021, TX USA). An *a-priori* decision was made to interpret findings mainly on the basis of model estimates and their 95% CIs rather than assign ‘significance’ using an arbitrary p value cutoff of 0.05. All p-values are still highlighted throughout, however, for reference. We also did not take account of multiple testing given the high correlation between many of our outcome variables.

# SUPPLEMENTARY TABLES

## Supplementary Table 1: BCS70 Participant Characteristics

|  |  |  | N | % or M(SD) |
| --- | --- | --- | --- | --- |
| *Demographics* | | |  |  |
|  | Age (years) | | 8210 | 47 ± 1 |
|  | Sex (% female) | | 8210 | 52 |
|  | Ethnicity (% white) | | 7114 | 97 |
| *Early-Life Factors* | | |  |  |
|  | Childhood Cognition | | 8171 | 0.3(1.5) |
|  | Childhood SES (% Father’s Occupation) | | 8161 |  |
|  |  | Professional |  | 8 |
|  |  | Managerial and Technical |  | 22 |
|  |  | Non-manual |  | 10 |
|  |  | Manual |  | 43 |
|  |  | Partly Skilled |  | 12 |
|  |  | Unskilled |  | 5 |
| *Mid-Life Factors* | | |  |  |
|  | Cognition | | 8210 | 0(1) |
|  | Systolic Blood Pressure (mmHg) | | 7278 | 124(15) |
|  | Diastolic Blood Pressure (mmHg) | | 7278 | 77(11) |
|  | Current BMI (kg/m^2^) | | 7165 | 27.7(5.8) |
|  | Malaise score | | 7613 | 1.8(2.1) |
|  | Physical Activity hr/day | | 8373 | 0.85(0.43) |
|  | Adult Socioeconomic Status % | | 6924 |  |
|  | Higher managerial and administrative | |  | 51 |
|  | Lower managerial and administrative | |  | 23 |
|  | Intermediate occupations | |  | 27 |
|  | Smoking status % | | 8210 |  |
|  | Everyday | |  | 15 |
|  | Occasional | |  | 5 |
|  | Historical | |  | 32 |
|  | Never | |  | 48 |
|  | Highest Education % | | 7104 |  |
|  | Higher degree | |  | 3 |
|  | Degree, other degree level | |  | 21 |
|  | Diploma of HE | |  | 7 |
|  | 2 or more A-levels | |  | 5 |
|  | 1 A level or more than 1 AS level | |  | 2 |
|  | O levels, good GCSEs | |  | 31 |
|  | CSE 2-5, other Scottish school qualification | |  | 8 |
|  | Bad GCSEs | |  | 1 |
|  | None | |  | 23 |

Data are mean ± standard deviation (SD) or %. DBP, diastolic blood pressure; PP pulse pressure; MAP, mean arterial pressure; SBP, systolic blood pressure; Childhood Socioeconomic Status is based on the occupation of the father; Malaise is the total from a derived Malaise Score; Physical Activity was the mean activity time of moderate to vigorous intensity over the day(hours/day); Adult Socioeconomic Status is the current economic activity; Smoking is the current smoking status; Highest Education is the highest academic level attained by age 26.

## Supplementary Table 2: Correlations between outcome measures at age 45 in the Dunedin Study

|  | **BrainAGE** | **WMH** | **Retinal Calibers** | **IQ** |
| --- | --- | --- | --- | --- |
| **BrainAGE** | 1 |  |  |  |
| **WMH** | 0.11  0.001 | 1 |  |  |
| **Retinal Calibers** | -0.08  0.014 | -0.08  0.024 | 1 |  |
| **IQ** | -0.20  <0.001 | -0.15  <0.001 | 0.04  0.293 | 1 |

Data represent Pearson correlation coefficients (top row of cell) and p-values (bottom row of cell), respectively. BrainAGE (difference between chronological age and age predicted from machine-learning models of brain-imaging data); WMH, white matter hyperintensities; IQ, intelligence quotient.

## Supplementary Table 3: Associations between Cumulative Systolic Blood Pressure Exposures during Childhood/Adolescence and Young Adulthood/Midlife with Midlife Brain Health and Cognitive Ability in the Dunedin Study

|  | **Model 1** | | | **Model 2** | | | **Model 3** | | | **Model 4** | | |
| --- | --- | --- | --- | --- | --- | --- | --- | --- | --- | --- | --- | --- |
|  | **β** | **95% CI** | **p-value** | **β** | **95% CI** | **p-value** | **β** | **95% CI** | **p-value** | **β** | **95% CI** | **p-value** |
| **Brain Age** | | | | | | | | | | | | |
| AUC, 7-26 | -0.06 | (-0.16, 0.04) | 0.251 | -0.02 | (-0.12, 0.08) | 0.690 | -0.02 | (-0.12, 0.08) | 0.732 | 0.01 | (-0.1, 0.11) | 0.885 |
| AUC, 26-45 | 0.07 | (-0.03, 0.17) | 0.171 | **0.12** | **(0.01, 0.23)** | **0.028** | **0.12** | **(0.01, 0.22)** | **0.025** | 0.01 | (-0.13, 0.15) | 0.885 |
| Systolic 45 |  |  |  |  |  |  |  |  |  | **0.12** | **(0.01, 0.23)** | **0.028** |
|  |  |  |  |  |  |  |  |  |  |  |  |  |
| **White Matter Hyperintensities** | | | | | | | | | | | | |
| AUC, 7-26 | **-0.11** | **(-0.21, -0.01)** | **0.032** | -0.10 | (-0.2, 0.01) | 0.065 | -0.09 | (-0.19, 0.01) | 0.088 | -0.07 | (-0.18, 0.03) | 0.164 |
| AUC, 25-45 | **0.12** | **(0.02, 0.22)** | **0.023** | 0.11 | (0.00, 0.21) | 0.054 | 0.10 | (0.00, 0.21) | 0.057 | 0.04 | (-0.1, 0.19) | 0.574 |
| Systolic 45 |  |  |  |  |  |  |  |  |  | 0.07 | (-0.04, 0.18) | 0.222 |
|  |  |  |  |  |  |  |  |  |  |  |  |  |
| **Retinal Arteriolar Caliber** | | | | | | | | | | | | |
| AUC, 7-26 | **0.11** | **(0.02, 0.21)** | **0.020** | 0.08 | (-0.02, 0.17) | 0.115 | 0.08 | (-0.02, 0.17) | 0.118 | 0.03 | (-0.07, 0.13) | 0.553 |
| AUC, 26-45 | **-0.36** | **(-0.46, -0.27)** | **< 0.001** | **-0.37** | **(-0.47, -0.27)** | **< 0.001** | **-0.37** | **(-0.47, -0.27)** | **< 0.001** | **-0.16** | **(-0.3, -0.02)** | **0.021** |
| Systolic 45 |  |  |  |  |  |  |  |  |  | **-0.23** | **(-0.34, -0.13)** | **< 0.001** |
|  |  |  |  |  |  |  |  |  |  |  |  |  |
| **IQ** | | | | | | | | | | | | |
| AUC, 7-26 | 0.01 | (-0.09, 0.11) | 0.877 | -0.01 | (-0.11, 0.09) | 0.892 | -0.03 | (-0.09, 0.03) | 0.361 | -0.03 | (-0.09, 0.03) | 0.302 |
| AUC, 26-45 | -0.02 | (-0.12, 0.07) | 0.632 | 0.00 | (-0.10, 0.11) | 0.944 | 0.01 | (-0.05, 0.08) | 0.688 | 0.03 | (-0.05, 0.12) | 0.459 |
| Systolic 45 |  |  |  |  |  |  |  |  |  | -0.02 | (-0.09, 0.04) | 0.513 |

Data are displayed as effect estimates, 95% Confidence Intervals (CI) and p-values obtained from multivariable linear regression models with the following adjustments: Model 1 = No adjustment; Model 2 = Adjustment for sex, BMI; Model 3 = Adjustment for sex, BMI, Childhood IQ, Education; Model 4 = Adjustment for sex, BMI, Childhood IQ, Education, Current Diastolic BP. AUC, area under the curve; BMI, body mass index; BP, blood pressure; IQ, intelligence quotient.

## Supplementary Table 4: Associations between Cumulative Diastolic Blood Pressure Exposures during Childhood/Adolescence and Young Adulthood/Midlife with Midlife Brain Health and Cognitive Ability in the Dunedin Study

|  |  | **Model 1** | | | **Model 2** | | | **Model 3** | | | **Model 4** | | |
| --- | --- | --- | --- | --- | --- | --- | --- | --- | --- | --- | --- | --- | --- |
|  |  | **β** | **95% CI** | **p-value** | **β** | **95% CI** | **p-value** | **β** | **95% CI** | **p-value** | **β** | **95% CI** | **p-value** |
| **Brain Age** | | | | | | | | | | | | | |
|  | AUC, 7-26 | 0.01 | (-0.07, 0.09) | 0.800 | -0.02 | (-0.10, 0.06) | 0.571 | -0.02 | (-0.10, 0.06) | 0.681 | -0.01 | (-0.09, 0.07) | 0.755 |
|  | AUC, 26-45 | 0.07 | (-0.01, 0.15) | 0.102 | **0.15** | **(0.06, 0.24)** | **0.001** | **0.14** | **(0.06, 0.23)** | **0.001** | **0.12** | **(0.00, 0.23)** | **0.043** |
|  | Diastolic 45 |  |  |  |  |  |  |  |  |  | 0.03 | (-0.07, 0.14) | 0.513 |
|  |  |  |  |  |  |  |  |  |  |  |  |  |  |
| **White Matter Hyperintensities** | | | | | | | | | | | | | |
|  | AUC, 7-26 | -0.07 | (-0.15, 0.01) | 0.089 | -0.07 | (-0.16, 0.01) | 0.082 | -0.07 | (-0.15, 0.01) | 0.098 | -0.06 | (-0.14, 0.02) | 0.163 |
|  | AUC, 26-45 | **0.09** | **(0.01, 0.17)** | **0.034** | **0.09** | **(0.00, 0.18)** | **0.042** | **0.09** | **(0.00, 0.18)** | **0.040** | 0.03 | (-0.09, 0.14) | 0.643 |
|  | Diastolic 45 |  |  |  |  |  |  |  |  |  | 0.09 | (-0.01, 0.20) | 0.083 |
|  |  |  |  |  |  |  |  |  |  |  |  |  |  |
| **Retinal Arteriolar Caliber** | | | | | | | | | | | | | |
|  | AUC, 7-26 | -0.02 | (-0.09, 0.05) | 0.601 | -0.01 | (-0.09, 0.06) | 0.766 | -0.01 | (-0.09, 0.06) | 0.752 | -0.04 | (-0.11, 0.04) | 0.300 |
|  | AUC, 26-45 | **-0.32** | **(-0.40, -0.25)** | **< 0.001** | **-0.34** | **(-0.42, -0.26)** | **< 0.001** | **-0.34** | **(-0.42, -0.26)** | **< 0.001** | **-0.17** | **(-0.27, -0.06)** | **0.003** |
|  | Diastolic 45 |  |  |  |  |  |  |  |  |  | **-0.24** | **(-0.34, -0.14)** | **< 0.001** |
|  |  |  |  |  |  |  |  |  |  |  |  |  |  |
| **IQ** | | | | | | | | | | | | | |
|  | AUC, 7-26 | 0.02 | (-0.05, 0.10) | 0.562 | 0.02 | (-0.06, 0.10) | 0.654 | -0.02 | (-0.07, 0.03) | 0.454 | -0.02 | (-0.07, 0.03) | 0.382 |
|  | AUC, 26-45 | -0.06 | (-0.14, 0.02) | 0.148 | -0.04 | (-0.13, 0.04) | 0.322 | -0.01 | (-0.06, 0.05) | 0.814 | 0.01 | (-0.06, 0.08) | 0.694 |
|  | Diastolic 45 |  |  |  |  |  |  |  |  |  | -0.03 | (-0.09, 0.03) | 0.380 |

Data are displayed as effect estimates, 95% Confidence Intervals (CI) and p-values obtained from multivariable linear regression models with the following adjustments: Model 1 = No adjustment; Model 2 = Adjustment for sex, BMI; Model 3 = Adjustment for sex, BMI, Childhood IQ, Education; Model 4 = Adjustment for sex, BMI, Childhood IQ, Education, Current Diastolic BP. AUC, area under the curve; BMI, body mass index; BP, blood pressure; IQ, intelligence quotient.

## Supplementary Table 5: Associations between blood pressure measured in childhood, adolescence, and midlife and cognitive function measured in midlife in BCS70

| **Standardised Beta (95% Confidence Intervals)** | | | | | | |
| --- | --- | --- | --- | --- | --- | --- |
|  | SBP | | | DBP | | |
|  | β | 95%CI | p | β | 95%CI | p |
| **Age 10** |  |  |  |  |  |  |
| Model 1 | -0.02 | (-0.06, 0.01) | 0.154 | -0.03 | (-0.06, 0.00) | 0.081 |
| Model 2 | -0.02 | (-0.05, 0.01) | 0.199 | -0.03 | (-0.06, 0.00) | 0.092 |
| Model 3 | -0.02 | (-0.05, 0.01) | 0.229 | -0.02 | (-0.05, 0.01) | 0.125 |
| **Age 16** |  |  |  |  |  |  |
| Model 1 | 0.00 | (-0.05, 0.04) | 0.931 | 0.00 | (-0.04, 0.05) | 0.899 |
| Model 2 | -0.02 | (-0.06, 0.02) | 0.370 | -0.01 | (-0.05, 0.03) | 0.655 |
| Model 3 | -0.01 | (-0.06, 0.03) | 0.500 | -0.01 | (-0.05, 0.03) | 0.698 |
| **Age 46** |  |  |  |  |  |  |
| Model 1 | **-0.09** | **(-0.12, -0.05)** | **<0.001** | **-0.08** | **(-0.11, -0.05)** | **<0.001** |
| Model 2 | **-0.04** | **(-0.07, -0.01)** | **0.016** | **-0.04** | **(-0.07, -0.01)** | **0.005** |
| Model 3 | **-0.03** | **(-0.06, 0.00)** | **0.047** | **-0.04** | **(-0.07, -0.01)** | **0.018** |

Data are displayed as effect estimates, 95% Confidence Intervals (CI) and p-values obtained from multivariable linear regression models with the following adjustments: Model 1 = Unadjusted; Model 2 = Model 1 + adjustment sex and for early life factors (childhood cognitive ability, childhood overcrowding and childhood socioeconomic status); Model 3 = Model 2 + further adjustment for mid-life factors (adult socioeconomic status, highest adult education, smoking status, malaise, and physical activity).

# SUPPLEMENTARY FIGURE

## Supplementary Figure 1 - Attrition analyses in the Dunedin Study


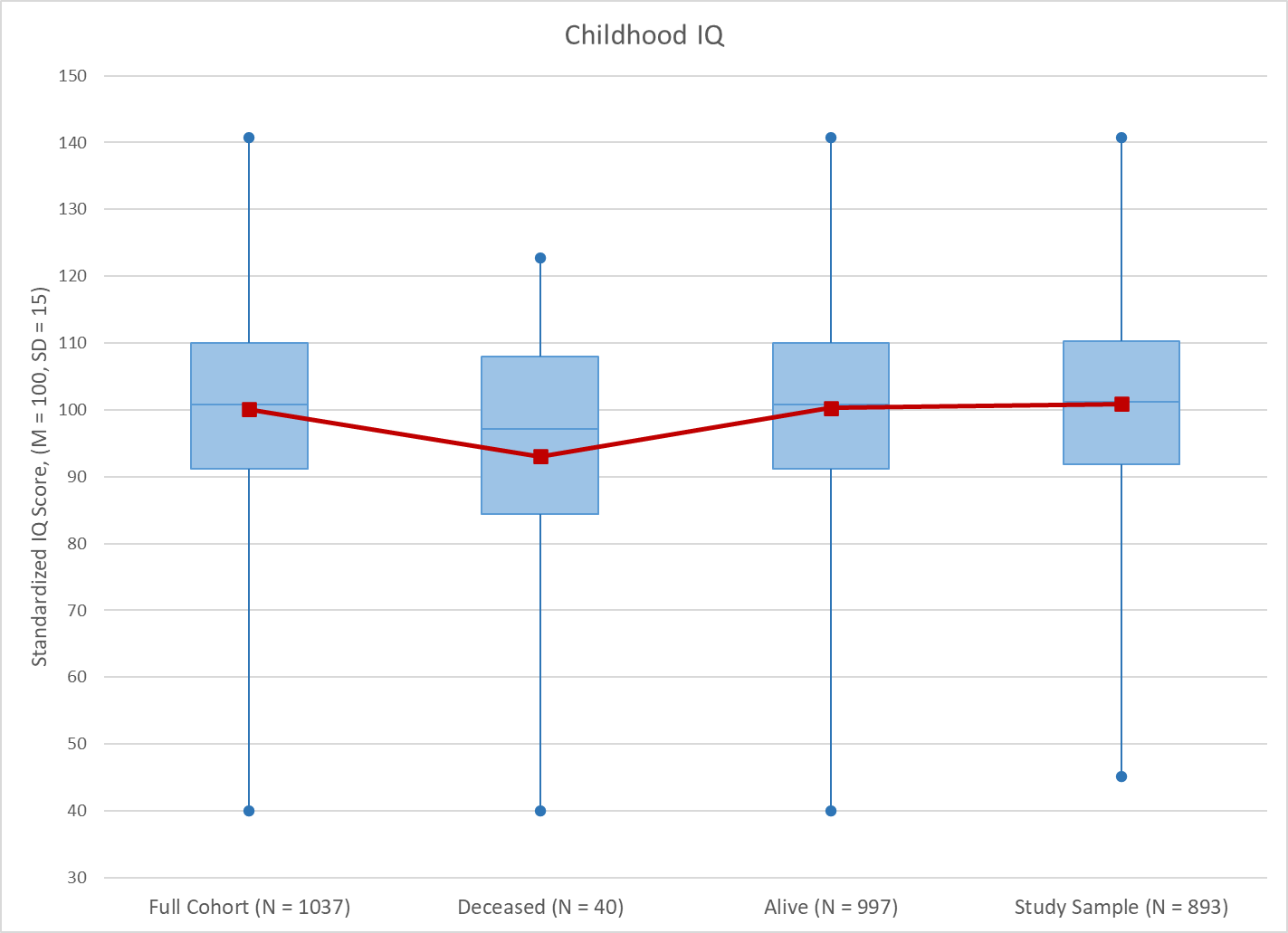


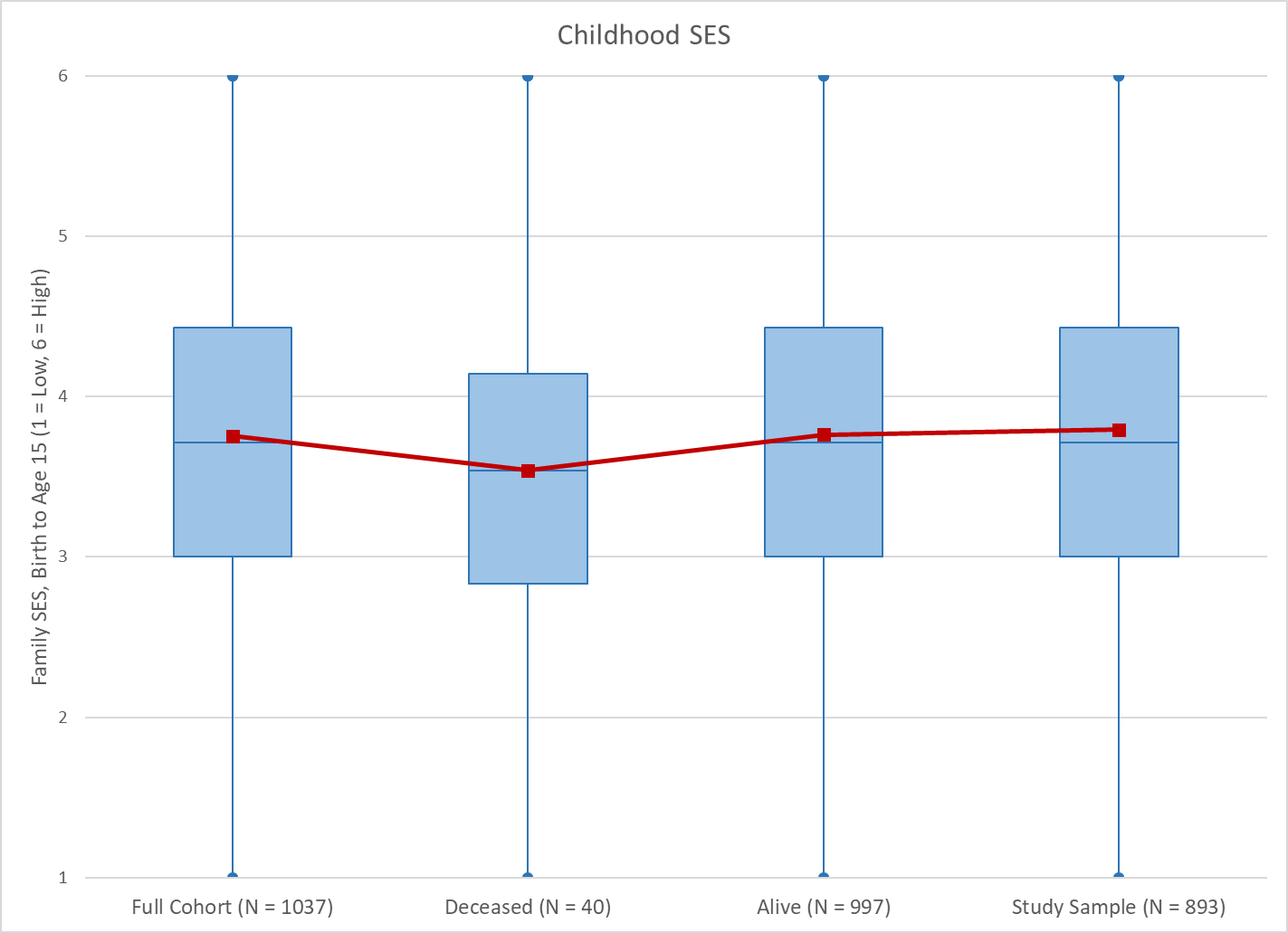


Supplementary Figure 1 shows results from an attrition analysis of the Dunedin Cohort at age 45. We report childhood IQ and childhood SES because they are known to be strong predictors of late-life health outcomes, as shown by many cohort studies from many nations. Childhood IQ and childhood SES separately predict health and social outcomes in adulthood, and these outcomes include physical functions, cognitive decline, mental health, inflammation, metabolic syndrome, disease incidence, dementia, mortality, and also neuroimaging-based, genomic, and epigenetic indicators of health. Based on the literature, we report three groups: Study members who died before age 45 and thus could not have taken part in data collection, Study members who were alive and thus could take part, and Study members included in the analyses reported here. We compared these three groups to the original birth cohort. The figures show that the small group of SM’s who had died before age 45 had significantly lower mean childhood IQ on average as a group, and somewhat lower mean childhood SES. Some of the early deaths were Dunedin Study members who had more disadvantages in their lives leading to poorer health and increased risk of early mortality. Study members who died of childhood diseases may have been already unwell at the time of IQ testing, which could have lowered their scores. However, cohort members who are still alive and cohort members who took part in data collection did not differ from the full original cohort on their mean childhood IQ and SES; they still represent population variation on these key health risk factors. Data within the figure represents planned t-tests. A significant mean difference was observed for childhood IQ between those alive vs deceased, t(36.28) = 2.09, p = .044 (Satterthwaite). Individuals who were deceased prior to the Phase 45 assessment had significantly lower IQ, M = 93.02, SD = 14.5, 95% CI: 86.02 – 100.03, than individuals who were still alive at Phase 45, M = 100.31, SD = 20.7, 95% CI: 99.40 – 101.22. Boxes indicate median with interquartile range (25th to 75th %) and error bars indicate ranges.

# SUPPLEMENTARY REFERENCE

1. Sullivan A, Brown M, Hamer M, Ploubidis GB. Cohort Profile Update: The 1970 British Cohort Study (BCS70). International Journal of Epidemiology. 2023;52:e179–e186.
